# 

**Published:** 1794

**Authors:** 


### CONTENTS.

Page

I. *AN Account of two Cases of Popliteal Aneurism. By Mr.* Thompson Forster, *Surgeon on the Staff of the Army, and Surgeon to Guy's Hospital.* — — 1

II. *An Account of the good Effects of Opium in the Case of a Person poisoned by Digitalis. By* Thomas Beddoes, *M. D.* 17

III. *Some Observations on the Diseases that occurred on Board the Ship Europa, in the Service of the Hon. East India Company, during a Voyage from England to and from Madrass and Bengal. By Mr* John Watson, *late Surgeon of the said Ship, and now Surgeon at Wellingborough, in Northamptonshire.* — — — 20

IV. *Case of a compound Dislocation of the Tibia and Fibula, accompanied with a Fracture and Loss of a considerable Portion of the Astragalus, and likewise with a Fracture of the Thigh Bone; with Re-*

*marks.*

Page

*marks. By Mr.* James Rumfey, *Surgeon at Amerfham in Buckinghamfhire.* — 44

V. *A Cafe of violent Diftortion of the Foot, occafioned by a Rotation of the Aftragalus, in confequence of a Fall, and accompanied with a Laceration of the Integuments at the outer Ancle, and Expofure of a Portion of the Fibula. By Mr.* William Guy, *Surgeon at Chichefter.* — — 54

VI. *Cafes of the Urticaria or Nettle Rafh, with Obfervations; By* T. M. Winterbottom, *M. D. Phyfician to the Settlement at Sierra Leone.* — — — 57

VII. *An Account of the Effects of Vitriolic Æther in a Cafe of fpafmodic Affection of the Stomach; and in two Cafes of Intermittent Fever. By Mr.* William Davidfon, *Apothecary in London.* — — 68

VIII. *An Account of the poifonous Effects of the Seeds of the Datura Stramonium* Linn. *By Mr.* James Johnfon, *Surgeon at Lancafter.* — — — — 78

IX. *A Cafe of Hydrophobia. By Mr.* Richard Simmons, *Surgeon to the Britifh Lying-in Hofpital.* — — — 87

X. *An Account of a Child born without Organs of Generation. By Mr.* Edward Ford,

Page

Ford, *F. A. S. Surgeon to the Westminster General Dispensary.* — — — 92

XI. *Case of Apoplexy in a pregnant Woman; with Observations. By Mr.* Philip Williams, *Surgeon at Rugby in Warwickshire.* 96

XII. *Description of Kilburn Wells, and Analysis of their Water. By Mr.* Joh. Godfr. Schmeisser. *From the Philosophical Transactions of the Royal Society of London.* — — — 100

XIII. *An Account of the remarkable Effects of a Shipwreck on the Mariners; with Experiments and Observations on the Influence of Immersion in fresh and salt Water, hot and cold, on the Powers of the living Body. By* James Currie, *of Liverpool, M. D. Fellow of the Royal College of Physicians at Edinburgh. From the same Work.* — — 103

XIV. *An Account of the Quassia Polygama, or Bitter-wood of Jamaica; and of the Cinchona Brachycarpa, a new Species of Jesuit's Bark found in the same Island. By Mr.* John Lindsay, *Surgeon in Westmoreland, Jamaica. From the Transactions of the Royal Society of Edinburgh.* — 140

XV. *Extract of a Letter from the Re-*

Page
*verend* Charles Perceval *to* Robert Perceval, *M. D. and M. R. I. A. From the Tranfactions of the Royal Irifh Academy.* — — — 157
XVI. *An Attempt to determine with Precifion fuch Injuries of the Head as neceffarily require the Operation of the Trephine. By* Sylvefter O'Halloran, *Efq. M. R. I. A. Honorary Member of the Royal College of Surgeons in Ireland, and Surgeon to the County of Limerick Hofpital. From the fame Work.* — — 161
XVII. *Account of a fiftulous Opening in the Stomach. By* George Burrowes, *M. D. M. R. I. A. From the fame Work.* 185
*Catalogue of Books.* — — — 190
*Index.* — — — — — 223

